# Role of Long Non-Coding RNAs and the Molecular Mechanisms Involved in Insulin Resistance

**DOI:** 10.3390/ijms22147256

**Published:** 2021-07-06

**Authors:** Vianet Argelia Tello-Flores, Fredy Omar Beltrán-Anaya, Marco Antonio Ramírez-Vargas, Brenda Ely Esteban-Casales, Napoleón Navarro-Tito, Luz del Carmen Alarcón-Romero, Carlos Aldair Luciano-Villa, Mónica Ramírez, Óscar del Moral-Hernández, Eugenia Flores-Alfaro

**Affiliations:** 1 Laboratorio de Epidemiología Clínica y Molecular, Facultad de Ciencias Químico-Biológicas, Universidad Autónoma de Guerrero, Chilpancingo 39087, GRO, Mexico; 07039464@uagro.mx (V.A.T.-F.); frebeltran@hotmail.com (F.O.B.-A.); marvar@uagro.mx (M.A.R.-V.); besteban@outlook.com (B.E.E.-C.); 13315606@uagro.mx (C.A.L.-V.); 2 Laboratorio de Biología Celular del Cáncer, Facultad de Ciencias Químico-Biológicas, Universidad Autónoma de Guerrero, Chilpancingo 39087, GRO, Mexico; nnavarro@uagro.mx; 3 Laboratorio de Citopatología e Histoquímica, Facultad de Ciencias Químico-Biológicas, Universidad Autónoma de Guerrero, Chilpancingo 39087, GRO, Mexico; lcalarcon@uagro.mx; 4 CONACyT, Facultad de Ciencias Químico-Biológicas, Universidad Autónoma de Guerrero, Chilpancingo 39087, GRO, Mexico; ruanomoni@hotmail.com; 5 Laboratorio de Virología, Facultad de Ciencias Químico-Biológicas, Universidad Autónoma de Guerrero, Chilpancingo 39087, GRO, Mexico

**Keywords:** long non-coding RNAs, insulin resistance, MALAT1, H19, MEG3, MIAT

## Abstract

Long non-coding RNAs (lncRNAs) are single-stranded RNA biomolecules with a length of >200 nt, and they are currently considered to be master regulators of many pathological processes. Recent publications have shown that lncRNAs play important roles in the pathogenesis and progression of insulin resistance (IR) and glucose homeostasis by regulating inflammatory and lipogenic processes. lncRNAs regulate gene expression by binding to other non-coding RNAs, mRNAs, proteins, and DNA. In recent years, several mechanisms have been reported to explain the key roles of lncRNAs in the development of IR, including metastasis-associated lung adenocarcinoma transcript 1 (MALAT1), imprinted maternal-ly expressed transcript (H19), maternally expressed gene 3 (MEG3), myocardial infarction-associated transcript (MIAT), and steroid receptor RNA activator (SRA), HOX transcript antisense RNA (HOTAIR), and downregulated Expression-Related Hexose/Glucose Transport Enhancer (DREH). LncRNAs participate in the regulation of lipid and carbohydrate metabolism, the inflammatory process, and oxidative stress through different pathways, such as cyclic adenosine monophosphate/protein kinase A (cAMP/PKA), phosphatidylinositol 3-kinase/protein kinase B (PI3K/AKT), polypyrimidine tract-binding protein 1/element-binding transcription factor 1c (PTBP1/SREBP-1c), AKT/nitric oxide synthase (eNOS), AKT/forkhead box O1 (FoxO1), and tumor necrosis factor-alpha (TNF-α)/c-Jun-N-terminal kinases (JNK). On the other hand, the mechanisms linked to the molecular, cellular, and biochemical actions of lncRNAs vary according to the tissue, biological species, and the severity of IR. Therefore, it is essential to elucidate the role of lncRNAs in the insulin signaling pathway and glucose and lipid metabolism. This review analyzes the function and molecular mechanisms of lncRNAs involved in the development of IR.

## 1. Introduction

Insulin resistance (IR) is a clinical condition defined as a reduced metabolic response to the action of insulin on target tissues, which are unable to coordinate a normal glucose-lowering response involving the suppression of endogenous glucose production, the suppression of lipolysis, cellular uptake of available plasma glucose, and glycogen synthesis. IR is a critical component of metabolic syndrome, obesity, and hyperlipidemia and is a risk factor for cardiovascular diseases (CVDs) and type 2 diabetes (T2D), especially when an insulin-resistant individual cannot secrete enough insulin from pancreatic β-cells [1,2]. Several mechanisms are involved in the etiology of IR, including mutations and post-translational modifications of the insulin receptor (IR), the insulin receptor substrate (IRS), and effector molecules located downstream. An essential factor that contributes to IR is the decreased phosphorylation of tyrosine residues (Tyr) in the β-subfraction of IR, which results in a decrease in its interaction with phosphatidylinositol 3-kinase (PI3K) and thus alters the phosphorylation and activation of protein kinase B (AKT) [3].

Several factors, such as proinflammatory cytokines, saturated fatty acids (SFAs), amino acids, endothelin 1, angiotensin II, and hyperinsulinemia, increase kinase activity [2,4]. Cellular senescence and chronic inflammation are known to be involved in the development of IR and T2D through both endocrine and metabolic effects, with the increased release of free fatty acids (FFAs) from adipocytes to the circulation. FFAs induce myocyte inflammation and metabolic dysfunction [5]. The accumulation of intramuscular lipids leads to insufficient oxidation of fatty acids by the mitochondria, which can be a causal factor in the development of IR. High concentrations of plasma FFAs are involved in the etiology of obesity-associated IR. In particular, SFAs such as palmitic acid markedly inhibit insulin-stimulated phosphorylation of IRS-1 and AKT, thereby inhibiting insulin-induced signaling. In addition, SFAs induce the expression of proinflammatory cytokines [6], an alteration that has also been associated with the development of IR.

Genome-wide association studies have identified approximately 88 loci associated with the risk of developing T2D. Most of the loci are related to insulin secretion and pancreatic β-cell function. Among the loci associated with IR are the peroxisome proliferator-activated receptor (*PPAR*) gamma, Kruppel-like factor 14 (*KLF14*), insulin receptor substrate 1 (*IRS1*), glucokinase regulator (*GCKR*), transcription factor 7-like 2 (*TCF7L2*), N-acetyltransferase (*NAT*) 1 and 2, transmembrane protein 163 (*TMEM163*), insulin-like growth factor (*IGF1*) 1, melanocortin 4 receptor (*MC4R*), transcription elongation regulator 1-like (*TCERG1L*), and sterol-C4-methyl oxidase-like (*SC4MOL*) [2,7,8]. In contrast, several miRNAs are involved in metabolic processes, including the regulation of insulin synthesis, secretion, and sensitivity. For instance, the overexpression of miR-29 led to IR, whereas miR-320 increased insulin sensitivity, miR-30d inhibited insulin expression, and miR-133 and miR-223 regulated glucose transporter type 4 (GLUT4) expression [9]. Other studies have reported associations between IR and let-7b, miR-144-5p, miR-34a, miR-532-5p [10], miR-27a [11], miR-34a, miR-592, and miR-194 [12].

Recently, the relationship between different long non-coding RNAs (lncRNAs) and the development and progression of metabolic diseases, including metabolic syndrome, liver diseases, obesity, IR, and diabetes, has been reported. However, the functional role of lncRNAs in the molecular mechanisms involved in IR development remains largely unknown. The aim of this review was to analyze the function and molecular mechanisms of the main lncRNAs involved in IR development.

## 2. Metabolic Processes of Insulin Resistance

### 2.1. Lipocentric and Glucocentric Approach in IR

Two of the most popular theories about IR development are the lipocentric and glucocentric approaches. In the first approach, IR is a consequence of lipotoxic effects. Lipotoxicity is considered an intracellular over-accumulation of FFAs or their derivatives, such as diacylglycerol (DAG), ceramides, and long-chain fatty acyl-coenzyme A (CoA), in adipose, liver, and muscle tissues [6,13]. This over-accumulation results in the activation of phosphokinase C (PKC). This serine/threonine kinase increases the phosphorylation of serine residues of IRS, which leads to the negative regulation on phosphatidylinositol 3-kinase/protein kinase B (PI3K/AKT) signaling pathway [14,15].

Additionally, the over-accumulation of lipids in the cytoplasm generates endoplasmic reticulum stress with subsequent activation of the unfolded protein response (UPR). UPR can activate c-Jun-N-terminal kinases (JNKs), serine/threonine kinases that promote the negative regulation of the insulin receptor (INSR) and IRS by phosphorylating serine residues [16]. Therefore, JNK may promote IR via the phosphorylation of IRS molecules. In addition, JNK promotes metabolic inflammation, adiposity, and negative regulation of *PPARα* and fibroblast growth factor 21 (*FGF21*). PPARα increases the transcription of genes related to the oxidative metabolism of fatty acids and the target gene *FGF21*, resulting in an increase in the hormone FGF21; this hormone promotes insulin sensitivity in the liver. For this reason, the inhibition of the PPARα–FGF21 axis by JNK is a positive feedback cycle in the development of IR [17]. Moreover, a high intake of SFAs has been reported to induce oxidation, inflammation, and impairment of hepatic insulin signaling in the liver tissue [18]. The molecular mechanism of SFAs contributes to the negative regulation of insulin signaling and activates proinflammatory pathways; SFAs activate toll-like receptor 4 (TLR4), which is expressed on immune cells, in white adipose tissue, and in the liver, to stimulate downstream proinflammatory processes [13].

On the other hand, in the glucocentric approach, IR development is linked to recurrent states of hyperglycemia that generate advanced glycation end products (AGEs), which exert pathogenic effects through interaction with cell surface receptors [19]. AGEs are involved in IR through direct and indirect actions of several pathways, including the activation of protein kinase C (PKC)-α, changes in the PI3K and mitogen-activated protein kinase (MAPK) insulin signaling pathways with opposing protective effects by sirtuin 1 (SIRT1), and the induction of oxidative stress and inflammation [20,21].

### 2.2. Causal Link between Oxidative Stress, Inflammation, and Insulin Resistance

Hyperglycemia contributes to increased reactive oxygen species (ROS) production by activating the PKC pathway, increasing hexosamine pathway flux, and promoting AGE production. Oxidative stress leads to altered glucose uptake in skeletal muscle and adipose tissue and decreases insulin secretion from β-cells, which can cause IR. ROS accumulation leads to non-specific oxidative damage to DNA, proteins, and lipids and consequently to cell death. Oxidative stress activates several signaling pathways, such as inhibitor of nuclear factor kappa/nuclear factor kappa B (IKKB/NF-κB) and JNK [22,23,24]. Additionally, hyperglycemia leads to the upregulation of proinflammatory factors, which induces endothelial dysfunction, leukocyte adhesion, and IR. Most cytokines and inflammatory mediators, such as tumor necrosis factor-alpha (TNF-α), monocyte chemoattractant protein-1 (MCP-1), C-reactive protein, and interleukins (IL), are upregulated in IR or high-glucose conditions [23,25]. TNF-α itself impairs insulin signaling via serine phosphorylation of IRS-1, leading to its degradation, and the translocation of GLUT4 is reduced.

Activation of IKKB/NF-κB and JNK pathways is a possible link between inflammation and IR [17,23]. The events that cause FFA-induced hepatic IR have been observed to occur via activation of PKC-δ and nicotinamide adenine dinucleotide phosphate oxidase (NADPH). These actions induce oxidative stress, leading to IKKδ- and JNK-mediated serine phosphorylation of IRS and the subsequent impairment of hepatic insulin signaling [6,26]. As previously described, the processes involved in the development of IR lead to the production of ROS, AGEs, and proinflammatory factors, which, in turn, will lead to other metabolic alterations, such as endothelial dysfunction and IR, and to the increased production of these molecules. Therefore, an unstoppable vicious cycle occurs if the necessary preventive measures are not implemented to reduce these alterations, which, of course, will lead to the generation of comorbidities associated with IR.

## 3. lncRNAs in Insulin Resistance

lncRNAs have been described as having an essential role in the molecular mechanisms involved in IR. They are single-stranded nucleotide sequences, ranging from approximately 200 bp to 10 kb in length, and are the most abundant non-coding RNAs in the human genome compared to microRNAs [27,28]. lncRNAs are usually polyadenylated and can have a multi-exonic structure. However, most of them are not translated into proteins, and it has been recently reported that some lncRNAs can encode small peptides. The majority of lncRNAs are transcribed by RNA polymerase II and often exhibit alternative splicing [29,30]. lncRNAs are classified by their genomic locations into (1) long intergenic ncRNAs (lincRNAs), which are located between protein-coding genes, (2) intronic lncRNAs, (3) sense lncRNAs, which are found on the sense strand of protein-coding genes and may contain exons from these genes, (4) antisense lncRNAs, which are located on the antisense strand, and (5) enhancer RNAs (eRNAs), which are produced from the bidirectional transcription of enhancer regions [29].

The subcellular localization of lncRNAs, either in the nucleus or in the cytoplasm, provides an indication of its function. Many lncRNAs are involved in gene regulatory processes at the nuclear level, including the repression or activation of transcription, activation or repression of multiple loci through chromatin remodeling, and epigenetic gene regulatory processes [31]. Additionally, lncRNAs can regulate gene expression post-transcriptionally by modulating the translation or stability of partially complementary mRNAs and interfering with RNA-binding proteins to influence splicing and translation. Competing endogenous RNAs (ceRNA) and circular RNAs are stable lncRNAs that accumulate in the cell and modulate gene expression by acting as decoys or sponges for microRNAs. Finally, some lncRNAs function post-translationally to control protein turnover by facilitating ubiquitination [32]. However, the specific characterization of lncRNA subcellular localization in IR models has focused on the endogenous competitor function and its localization in the cytoplasm, whereas the characterization of lncRNAs at the nuclear level is lacking.

At present, the GENCODE database (v.37) identifies 17,957 genes as lncRNAs [33]. Several lncRNAs have been confirmed to be related to IR, such as metastasis-associated lung adenocarcinoma transcript 1 (MALAT1), imprinted maternally expressed transcript (H19), maternally expressed gene 3 (MEG3), myocardial infarction-associated transcript (MIAT), and steroid receptor RNA activator (SRA). However, there is still much to learn about the function, molecular processes involved, and expression of these non-coding RNAs in IR.

### 3.1. Metastasis-Associated Lung Adenocarcinoma Transcript 1 (MALAT1)

MALAT1 is a widely studied lncRNA; it is also known as nuclear-enriched abundant transcript 2 (NEAT2), HCN, LINC00047, NCRN00047, and PRO2853. In cancer, it has been associated with the transcriptional and post-transcriptional regulation of numerous genes involved in the regulation of the cell cycle, metastasis, cell migration, and resistance to antineoplastic drugs and with the prognosis and development of cancer [34]. Moreover, it has been reported that MALAT1 is related to the etiology and progression of metabolic diseases, such as T2D [35], metabolic syndrome [36], diabetic retinopathy [37], CVD [38], diabetic nephropathy [39], and gestational diabetes [40].

The MALAT1 gene is located on chromosome 11q13.1 and has an approximate length of 6.7 kb, and the 3’ region lacks a poly-A tail. The 3’-end of MALAT1 forms a unique triple-helix structure that protects it from 3’-5’ exonucleases. MALAT1 is highly expressed to a similar or even higher degree than some proteins, such as β-actin and D-glyceraldehyde-3-phosphate dehydrogenase. MALAT1 is predominantly located in the nucleus; however, a small proportion of this molecule can be found in the cytoplasm [34,39,41,42].

**Figure 1:**
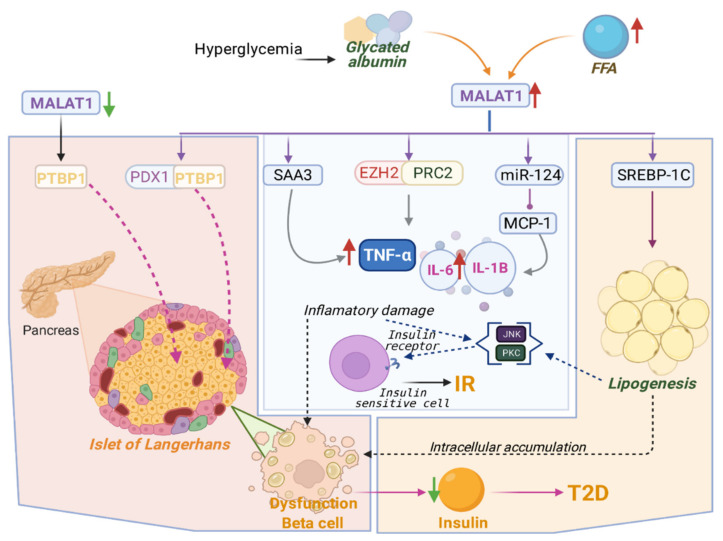
Role of metastasis-associated lung adenocarcinoma transcript 1 (MALAT1) in the molecular mechanisms of insulin resistance. The downregulation of MALAT1 affects the modulation of polypyrimidine tract-binding protein 1 (PTBP1), demonstrating that it contributes to the dysfunction of pancreatic β-cells and the development of type 2 diabetes (T2D). In states of hyperglycemia and high levels of free fatty acids (FFAs), MALAT1 is overexpressed through the activation of the PDX1/MALAT1/PTBP1 axis and indirectly promotes the overexpression of tumor necrosis factor-alpha (TNF-α), interleukins (IL)-6, and IL-1β when bound to polycomb repressive complex 2 (PRC2), inhibiting the expression of anti-inflammatory genes. Glycated albumin induces monocyte chemoattractant protein-1 (MCP-1) expression, which depends on the MALAT1/miR-124 axis. MALAT1 directly interacts with SREBP-1c, inhibiting its ubiquitination and increasing its activity, which leads to the expression of its target lipogenic genes, thus inducing intracellular lipid accumulation and IR. Increased transcription of proinflammatory and lipogenic genes leads to the activation of threonine/serine kinases c-Jun-N-terminal kinases (JNK) and phosphokinase C (PKC), which are associated with the negative regulation of insulin signaling.

The function of MALAT1 occurs inside cells, primarily in the nucleus. In a hyperglycemic state, the global inhibition of DNA methyltransferase (DNMT) activity induces significant dysregulation in the expression of MALAT1, probably due to the hypomethylation of CpG islands in the MALAT1 promoter [43]. Additionally, MALAT1 downregulation may contribute to the dysfunction of pancreatic β-cells and the development of T2D via direct interaction and modulation of polypyrimidine tract-binding protein 1 (PTBP1), demonstrating the scaffolding activity of MALAT1. Moreover, hyperglycemia and elevated levels of FFAs also induce an increase in the transcriptional factor pancreatic and duodenal homeobox 1 (PDX1), which, in turn, increases the transcription of MALAT1 and can induce β-cell dysfunction through the PDX1/MALAT1/PTBP1 pathway [44]. These events increase the transcription of *MALAT1* and its levels in the nucleus. MALAT1 indirectly promotes the overexpression of the proinflammatory genes *TNF-α*, *IL-6*, and *IL-1β*. This event occurs when MALAT1 is bound to enhancer of zeste polycomb homolog 2 (EZH2), which is the catalytic subunit of polycomb repressive complex 2 (PRC2), leading to histone methylation of anti-inflammatory genes, transcriptional repression, and therefore, to increased transcription of proinflammatory genes [43]. As mentioned earlier, the overexpression of proinflammatory genes results in a systemic inflammatory state with the subsequent activation of serine/threonine kinases such as JNK; the activation of JNK results in the negative feedback of the PI3K–AKT pathway activated by insulin through of mechanism above mentioned (Figure 1).

Dong et al. reported that glycated albumin induced *MCP-1* expression in retinal microglia and observed that its expression was dependent on the MALAT1/miR-124 axis, which has been previously demonstrated to have an important relationship with inflammatory processes in diabetic mice and cell cultures exposed to high concentrations of glucose [45]. Another proinflammatory mechanism due to hyperglycemia mediated by MALAT1 is the upregulation of serum amyloid antigen 3 (SAA3), which stimulates the production of proinflammatory cytokines TNF-α and IL-6, promoting endothelial inflammation, which may lead to CVD [46]. Furthermore, in hepatocytes under fatty acid overload conditions, MALAT1 directly interacts with sterol regulatory element-binding transcription factor 1c (SREBP-1c), an essential regulator of lipogenesis. This binding inhibits the ubiquitination of SREBP-1c, increasing SREBP-1c activity and the expression of its target lipogenic genes, which induces intracellular lipid accumulation and IR (Figure 1) [47].

As previously described, in a hyperglycemic state, MALAT1 is involved in the increased transcription of proinflammatory and lipogenic genes, leading to the activation of threonine/serine kinases JNK and PKC. These increases are associated with negative regulation in the insulin signaling pathway (Figure 1) [15]. However, more studies are necessary to establish the relation between MALAT1, JNK, and PKC. In addition, rigorous studies are required to evaluate the effects of the hyper-insulinemic state on MALAT1 activity.

### 3.2. Imprinted Maternally Expressed Transcript (H19)

H19, a 2.3 kb RNA, is highly expressed in the fetal stage and evolutionarily conserved. H19 plays an important role in embryonal development, mainly in mesoderm and endoderm-derived tissues. Its expression is strongly downregulated after birth, except in cardiac and skeletal muscle. This lncRNA is transcribed exclusively from the maternal allele and resides in the *H19-IGF2* locus, a highly conserved imprint located on human chromosome 11p15.5, where these two genes are reciprocally imprinted. The *H19* gene comprises five exons separated by small introns and produces a spliced, capped, and polyadenylated RNA that mainly resides in the cytoplasm [48,49]. The hypermethylation of the *H19* gene promoter and allele-specific methylation of the 3′ portion of *H19* may be related to changes in its expression [50].

Different studies have reported that H19 overexpression contributes to IR development; however, the results have been contradictory. An increase in the expression levels of H19 associated with decreased methylation at CpG sites and reduced folate levels has been reported in the liver tissue of patients with T2D [51]. Moreover, the binding of S-adenosylhomocysteine hydrolase (SAHH) to H19 has been shown to interfere with its ability to hydrolyze S-adenosylhomocysteine (SAH), which is a potent inhibitor of S-adenosylmethionine (SAM)-dependent DNMTs. This interaction leads to decreased DNMT3B-mediated methylation of different genomic loci, which could be associated with the development of various pathological conditions [52].

Under normal physiological conditions, hepatic glucose production (HGP) is controlled by glucagon (a positive regulator) and insulin (a negative regulator) in response to fasting or postprandial states, respectively. In the fasting state, glucagon promotes gluconeogenesis and glycogenolysis through the increased expression of phosphoenolpyruvate carboxykinase (PEPCK) and G6Pase. This effect is linked to the interaction of forkhead box O1 (FoxO1) and peroxisome proliferator-activated receptor-gamma coactivator-1 alpha (PGC1α). However, after a rise in the blood glucose level with subsequent hyperinsulinemia, insulin promotes the phosphorylation of FoxO1, leading to its nuclear exclusion, which results in a decrease in PEPCK and G6Pase expression and the subsequent inhibition of HGP [53].

**Figure 2:**
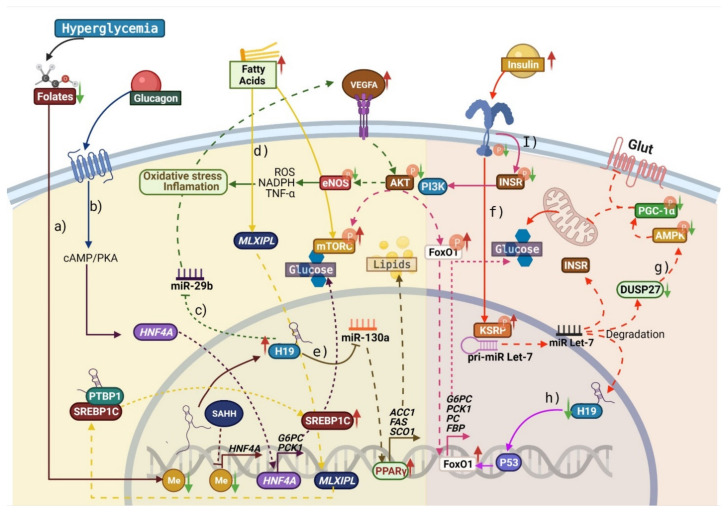
Imprinted maternally expressed transcript (H19) alterations and their molecular mechanisms. (**a**) The decrease in folates due to hyperglycemia leads to hypomethylation (Me) of the *H19* promoter, increasing its transcription. H19 inactivates S-adenosylhomocysteine hydrolase (SAHH) and inhibits hepatocyte nuclear factor 4 alpha (*HNF4A*) promoter methylation. (**b**) The glucagon-stimulated upregulation of H19 via the cyclic adenosine monophosphate/ protein kinase A (cAMP/PKA) axis induces nuclear translocation of HNF4A and activates the transcription of gluconeogenic genes glucose-6-phosphatase catalytic (*G6PC*) and phosphoenolpyruvate carboxykinase (*PCK*). (**c**) Inhibition of miR-29b by H19 increases vascular endothelial growth factor-A (VEGFA) expression, leading to decreased activation of protein kinase B (AKT) and nitric oxide synthase (eNOS), increasing reactive oxygen species (ROS) production and proinflammatory cytokines. (**d**) High levels of free fatty acids (FFAs) promote H19 overexpression, which induces increased mTOR complex 1 (mTORC) signaling, nuclear translocation of MLX-interacting protein-like (MLXIPL) and element-binding transcription factor 1c (SREBP-1c), and the interaction of polypyrimidine tract-binding protein 1 (PTBP1) with SREBP-1c, which in turn induces lipid accumulation. (**e**) The binding of H19 to miR-130a induces the nuclear translocation of proliferator-activated receptor gamma (PPARγ), which activates the transcription of lipogenic genes such as acetyl-CoA carboxylase 1 (ACC1), fatty acid synthase (FAS), and cytochrome C oxidase (SCO1), promoting the intracellular accumulation of lipids. (**f**) Hyperinsulinemia induces the activation of far upstream element-binding protein (KSRP), which forms a complex with primiR Let7, promoting its maturation to miR-Let7, which binds H19, insulin receptor (INSR), and dual-specificity phosphatase 27 (DUSP27), degrading them. (**g**) The decrease in DUSP27 reduces the activation of cAMP-activated protein kinase (AMPK) and peroxisome proliferator-activated receptor gamma coactivator 1-alpha (PGC-1α), which inhibits glucose uptake and mitochondrial biogenesis. (**h**) H19 downregulation induces the binding of p53 to the forkhead box O1 (*FoxO1*) promoter, increasing its transcription. (**I**) The insulin signaling pathway is deregulated, decreasing the phosphorylation of INSR and AKT by phosphatidylinositol 3-kinase (PI3K), which induces an increase in mTORC and FoxO1, promoting the translocation of FoxO1 to the nucleus and the transcription of gluconeogenic genes.

In this context, increased expression of H19 in the liver of diabetic mice is associated with increased HGP and glucose intolerance. In contrast, the loss of H19 function improves insulin sensitivity and suppresses HGP. H19 induces hypomethylation of the promoter of hepatocyte nuclear factor 4 alpha (*HNF4A*), increasing its expression and stimulating the expression of the gluconeogenic genes *PEPCK1* and glucose-6-phosphatase catalytic (*G6PC*), which reduces the ability of insulin to suppress HGP. Additionally, the glucagon-stimulated upregulation of H19 via the cyclic adenosine monophosphate (cAMP)/PKA pathway induces nuclear translocation of HNF4A and activates the transcription of the gluconeogenic genes *G6PC* and *PCK* and, as a result, HGP (Figure 2) [54].

In contrast, decreased H19 expression in IR or associated conditions has been reported in different studies. Gui et al. found that the expression of H19 was reduced in skeletal muscle samples of obese and diabetic mice (db/db mice, used as a model of IR). In addition, they used a model of H19 overexpression in db/db mice to assess its effect on glucose and lipid metabolism by measuring multiple metabolic parameters. They found that H19-overexpressing mice had significantly improved glucose tolerance and decreased serum levels of insulin, triglycerides (TG), and cholesterol, and decreased TG levels in skeletal muscle were identified. Additionally, the overexpression of H19 in muscle increased acetyl-CoA carboxylase 1 (ACC1) and cAMP-activated protein kinase (AMPK) phosphorylation and increased the expression of peroxisome proliferator-activated receptor gamma coactivator 1-alpha (PGC-1α), SIRT1, carnitine palmitoyltransferase 1b, and CD36, which suggests that H19 overexpression ameliorates glucose intolerance, dyslipidemia, and IR (Figure 2). Furthermore, using RNA pull-down and immunoprecipitation assays, the authors identified the interaction of H19 with heterogeneous nuclear ribonucleoprotein A1 (hnRNPA1), which has been reported to play an important role in glucose and lipid metabolism [55].

Gao et al. showed a double-negative feedback loop between the H19 and lethal-7 gene (*Let-7*) in muscle cells. Acute hyperinsulinemia enhances the activation of the PI3K/AKT pathway, promoting an increase in the biogenesis of miR-let-7, which binds to H19 for its degradation. Therefore, a decrease in H19 levels and higher bioavailability of miR-let-7 could increase glucose uptake. Moreover, miR-let-7-mediated repression of multiple components of the insulin-PI3K-mammalian target of rapamycin (mTOR) pathway, including INSR and IRS2, leads to impaired glucose tolerance [56]. Goyal et al. observed that H19 inhibition promoted an increase in the expression of gluconeogenic genes, which was associated with higher HGP and hyperglycemia and hyperinsulinemia in diabetic (db/db) mouse liver and HepG2 cells. They also identified that H19 downregulation was correlated with the enrichment of *G6PC*, *PCK1*, pyruvate carboxylase (*PC*), and fructose-1,6-biphosphatase (*FBP*) genes, which are involved in glycolysis and gluconeogenesis. In addition, H19 depletion in HepG2 cells increased the nuclear localization of FoxO1 (an essential transcriptional factor in the regulation of gluconeogenesis and in the insulin response in the liver), impaired insulin signaling, and increased gluconeogenesis, suggesting the functional relevance of decreased H19 levels in the pathophysiology of T2D [57]. Subsequently, the same working group demonstrated that the inhibition of H19 caused a significant increase in FoxO1 transcript and protein levels and impaired glucose, insulin, and pyruvate tolerance. Additionally, they identified that, in the absence of H19, p53 significantly occupied the FoxO1 promoter, suggesting that the inhibition of H19 promotes the transcription of *FoxO1* through p53. Furthermore, they demonstrated that the inhibition of H19 in vivo induced hyperglycemia and hyperinsulinemia and increased the transcription of hepatic gluconeogenic genes. These results suggest that H19 inhibition deregulates both circulatory and metabolic profiles and hepatic gluconeogenesis, possibly via the p53/FoxO1 axis (Figure 2) [58].

In another study, elevated H19 expression in the skeletal muscle of mice improved insulin sensitivity. In contrast, H19 knockout (KO) decreased insulin-stimulated glucose uptake in muscle cells; this observed effect could be explained by the disruption of the H19/dual-specificity phosphatase 27 (DUSP27)/AMPK axis. The authors suggested that a reduction in H19 results in the lower expression of DUSP27 with the subsequent reduction in the phosphorylation of AMPK (at Thr172) and ACC1 (at Ser79), resulting in an insulin-resistant state. Moreover, the overexpression of DUSP27 in H19 KO mice resulted in an increase in AMPK phosphorylation and insulin-stimulated glucose uptake similar to wild-type H19 mice and improved mitochondrial biogenesis, fatty acid biosynthesis, glucose uptake, and muscle insulin sensitivity (Figure 2) [59].

Non-alcoholic fatty liver disease, a consequence of the high production and accumulation of lipids in the liver, is considered a key factor in the development of IR and T2D [60]. A mechanism has been proposed in which H19 promotes hepatic lipid synthesis by binding to PTBP1, which interacts with SREBP-1c, allowing greater stability, increasing its cleavage and nuclear translocation, and improving its transcriptional activity [61]. Activation of PI3K/pyruvate dehydrogenase kinase 1/PKB is required for insulin to induce SREBP-1c expression in the liver, which, together with glucokinase (GK), exerts a synergistic effect on glycolytic and lipogenic genes. It has been proposed that hyperinsulinemia, which prevails in IR, could induce a decrease in IRS-2 content. This event, in turn, could account for the IR-mediated repression of gluconeogenic genes, whereas insulin could still activate lipogenic genes by IRS-1. It has been suggested that mTOR complex 1 (mTORC1) is involved in SREBP-1c expression. As mTORC1 integrates not only signals arising from insulin but also nutrients, it has been suggested that nutrient signals such as amino acids could be excessive in obesity and replace the deficient insulin signal, thus worsening IR. On the other hand, endoplasmic reticulum stress, which is present in the liver of IR rodents, has been shown to activate SREBP-1c, inducing IR through the activation of stress kinases as JNK [62].

High-fat diet (HFD)-induced H19 upregulation promotes MLX-interacting protein-like (MLXIPL) expression, a glucose-dependent lipogenic transcription factor, and promotes its nuclear translocation. In addition, H19 positively regulates mTORC1 signaling and the activation of the H19/PTBP1/SREBP-1c axis; together, these events induce lipid accumulation in hepatocytes [63]. On the other hand, Liu et al. showed that elevated H19 expression promotes hepatic steatosis via the miR-130a/PPARγ axis, which induces the transcription of lipogenic *SREBP-1c*, *ACC1*, and fatty acid synthase (*FAS*) [64]. As a result that oxidative stress and inflammation play an important role in T2D, endothelial cells were treated with glucose in various doses and intervals, resulting in significant dose- and time-dependent upregulation of H19, and vascular endothelial growth factor (VEGF)-A expression was also significantly upregulated. In contrast, after the administration of glucose, the expression of miR-29b was considerably downregulated, and a proinflammatory environment and oxidative stress were induced, which suggests that the H19/miR-29b/VEGFA pathway plays a role in hyperglycemic-induced endothelial dysfunction. Additionally, after H19 downregulation, the expression of proinflammatory cytokines, the production of ROS, and NADPH oxidase activity all decreased, while miR-29b expression and activation of the AKT/nitric oxide synthase (eNOS) pathway increased [65]. The participation of H19 in different metabolic pathways is summarized in Figure 2.

As a major lncRNA that has been widely studied in metabolic diseases, H19 has been proposed as a biomarker for the diagnosis of IR and T2D; however, at present, this is not possible due to the contradictory findings reported by different studies to date. In a study carried out on samples of subcutaneous (SAT) and visceral (VAT) adipose tissue from women, a significant decrease in the expression levels of H19 in SAT from women with obesity and IR was identified. Additionally, the expression levels of both ACC1 and FAS, two critical molecules required for fatty acid biogenesis, were elevated in obese patients. In addition, the expression of H19 was negatively correlated with FAS, suggesting the essential participation of H19 in fatty acid biogenesis [66]. On the other hand, recent studies have investigated H19 in non-invasive samples, such as blood plasma and serum. At least two studies have reported the elevated expression of H19 in blood plasma samples from diabetic patients compared to healthy patients [65,67]. In contrast, our group identified low H19 expression in blood serum samples from patients with metabolic syndrome [68]. The contradictory results for the expression of H19, as well as the variability in the clinical characteristics of the patients, such as the timing in the progression of IR or T2D, the metabolic control, and the treatment, could contribute to the variation in the expression of lncRNAs. This suggests that, for the diagnosis or prognosis of these diseases, a set of lncRNAs should be used as biomarkers, not only one of them.

### 3.3. Maternally Expressed 3 (MEG3)

The *MEG3* gene is located on chromosome 14q32.2, contains ten exons with an approximate length of 1700 nucleotides, is a maternally expressed imprinted gene with different isoforms generated by alternative splicing, and is expressed in many normal tissues. Several miRNAs have been shown to regulate the expression of *MEG3* at the post-transcriptional level. In turn, MEG3 acts as competing endogenous RNA for several miRNAs [69], including miR-466i-5p, miR-574-5p, and miR-770-5p. Interestingly, the genes targeted by miR-466i-5p, miR-574-5p, and miR-770-5p were identified as hub genes modulated by MEG3 in IR conditions; the target genes include prolyl 3-hydroxylase 2 (*LEPREL1*), integrin alpha M (*ITGAM*), and collagen type VI alpha chain (*COL6A2*), which are expressed in adipose tissue and involved in regulating inflammation, fibrosis, and insulin signaling, respectively [70].

Mondal et al. mapped MEG3 binding sites across the genome in BT-549 cells; they reported more than 6800 sites of union. A significant proportion of these sites are located distal to the promoter regions. The authors demonstrated that transforming growth factor-beta (TGF-β) pathway genes are direct targets of MEG3. These results are consistent with another investigation reporting that MEG3 expression was downregulated upon TGF-β1 treatment in human hepatic stellate cells and that its overexpression inhibited TGF-β1-stimulated cell proliferation and induced apoptosis [71]. In this regard, the participation of the TGF-β1 signaling pathway in the development of IR has been identified, either through the effect of miR335-5p on the inhibition of vasohibin-1 expression and stimulation of the TGF-β signaling pathway [72] or through the overexpression of tribbles (protein kinase) induced by TGF-β/glass bottom boat axis [73].

**Figure 3:**
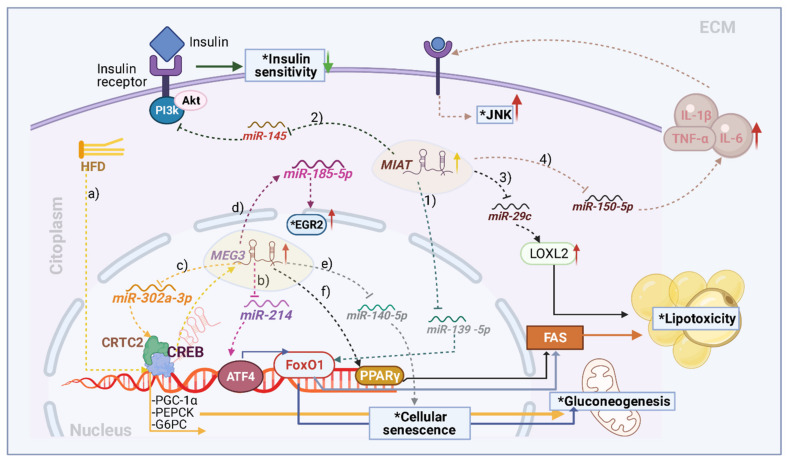
Molecular effects of maternally expressed gene 3 (MEG3) and myocardial infarction-associated transcript (MIAT) on insulin resistance. (**a**) High-fat diet (HFD)-induced cAMP response element-binding protein (CREB) activation promotes MEG3 expression. (**b**) MEG3 overexpression inhibits miR-214, promoting activating transcription factor 4 (ATF4) expression, activating forkhead box O1(FoxO1), and increasing fatty acid synthase (FAS), leading to a lipotoxic environment. (**c**) MEG3 inhibits the action of miR-302a-3p, which leads to the activation of the CREB-CREB-regulated transcriptional coactivator (CRTC2) complex, increasing their interaction with ATF4 and leading to the expression of gluconeogenic proteins. (**d**) MEG3 expression inhibits miR-185-5p, leading to early growth response protein-2 (EGR2) overexpression. (**e**) Inhibition of miR-140-5p by MEG3 promotes cellular senescence. (**f**) MEG3 overexpression increases proliferator-activated receptor gamma (PPARγ) expression and FAS activation, leading to the production of fatty acids and lipotoxic effects. Furthermore, (**1**) the overexpression of MIAT prevents the inhibition of FoxO1 by miR-139-5p, promoting FAS expression and raising the levels of FFAs. (**2**) MIAT inhibits miR-145, thereby inhibiting the phosphatidylinositol 3-kinase/protein kinase B (PI3K/AKT) pathway, which leads to a reduction in insulin sensitivity. (**3**) The inhibition of miR-29c by MIAT facilitates the expression and action of lysyl oxidase-like 2 (LOXL2), promoting a lipotoxic environment. (**4**) After the inhibition of miR-150-5p by MIAT, the levels of tumor necrosis factor-alpha (TNF-α), and interleukins 6, and 1β increase.

In a mouse model of HFD-induced IR, Chen et al. identified the overexpression of MEG3 and early growth response protein-2 (EGR2), while miR-185 was downregulated. In addition, MEG3 and miR-185-5 expression were negatively correlated, while MEG3 and EGR2 expression were positively correlated. Additionally, in HepG2 cells, the authors observed that elevated MEG3 expression aggravated palmitate-induced IR by regulating the miR-185-5p/EGR2 axis, suggesting that MEG3 may be a useful target in new therapeutic strategies for T2D (Figure 3) [74].

Zhu et al. reported that the expression of MEG3 was upregulated in HFD and obese (ob/ob) mice. Moreover, high fat enhanced MEG3 expression in hepatocytes through histone acetylation. MEG3 overexpression significantly increased FoxO1, G6PC, and PCK1 expression and hepatic gluconeogenesis and suppressed insulin-stimulated glycogen synthesis in primary hepatocytes, which led to increased hepatic IR [75]. Subsequently, the same group identified increased MEG3 expression and activating transcription factor 4 (ATF4) (a factor that regulates gluconeogenesis by affecting the transcriptional activity of FoxO1) and decreased miR-214 expression in liver tissue from HFD and ob/ob mice. Additionally, they showed that MEG3 knockdown upregulated miR-214 expression and downregulated ATF4 expression. These findings suggest that MEG3 functions as a ceRNA of miR-214 to upregulate ATF4 expression, leading to the activation of FoxO1 and FAS, which in turn promotes lipogenesis and IR [76]. In the same year, these researchers proposed that cAMP response element-binding protein (CREB) induces MEG3 upregulation and increases the hepatic gluconeogenic genes *PGC-1α*, *PEPCK*, and *G6PC* through the CREB–CREB-regulated transcriptional coactivator (CRTC2) complex, which contributes to the pathogenesis of hepatic gluconeogenesis, suggesting that MEG3 acts as a ceRNA to upregulate CRTC2 via miR-302a-3p (Figure 3) [77].

Furthermore, in a study carried out in samples of SAT and VAT adipose tissue from women, MEG3 expression in SAT had a significant relationship with obesity indices. In addition, MEG3 expression in SAT was positively correlated with PPARγ and FAS expression (Figure 3), suggesting an essential role for MEG3 in obesity-related conditions [66]. Cheng et al. identified an increase in the expression of MEG3 in endothelial cells isolated from white adipose tissue compared to those from the skeletal muscle or liver of obese mice. Additionally, they observed that MEG3 knockdown induced endothelial senescence in vitro and cellular senescence of hepatic endothelium, promoting obesity-induced IR in obese mice. Researchers have suggested that the manipulation of MEG3 expression may represent a novel approach to managing obesity-associated hepatic endothelial senescence and IR [78].

Additionally, significant MEG3 overexpression has been reported in patients with T2D, and this overexpression was positively correlated with poor glycemic control, IR, transcriptional markers of senescence, and inflammation [35]. Liu et al. reported that MEG expression was markedly downregulated, while miR-140-5p was upregulated in circulating endothelial progenitor cells (EPCs) of patients with metabolic syndrome. miR-140-5p is associated with cellular senescence. Moreover, treatment with pioglitazone significantly increased MEG3 expression and decreased miR-140-5p levels in circulating EPCs, suggesting that MEG3 acts as a ceRNA of miR-140-5p (Figure 3) [79].

### 3.4. Myocardial Infarction-Associated Transcript (MIAT)

MIAT is also known as Gomafu or retinal non-coding RNA 2; its gene is located on the short arm of human chromosome 22q12.1. MIAT contains multiple spliced exons, with a final transcript size of approximately 10,000 nucleotides, which is polyadenylated; it is not exported to the cytoplasm and is concentrated in the cell nucleus [80]. MIAT can bind to and sequester miRNAs via base pairing, functioning as a ceRNA to regulate mRNA expression. For example, MIAT inhibited high-mobility group box 1 (HMGB1) expression by competitively binding to miR-204-5p to regulate the injury of cerebral artery occlusion after cerebral ischemia in rats [81]. Some studies have identified relationships between MIAT and several diseases, such as schizophrenia, prostate cancer, lung cancer, diabetes, and complications of diabetes [82]. Significantly increased levels of MIAT have been reported in patients with T2D, possibly related to poor glycemic control, IR, cellular senescence, and inflammation [35]. Moreover, the plasma levels of MIAT were significantly increased in patients with acute myocardial infarction (AMI) [83]. Genetic variants in MIAT were initially recognized as a plausible biomarker linked to the development of AMI in the Japanese population [84].

As in most lncRNAs, the ceRNA function is a molecular mechanism through which MIAT exerts its pathogenic effects. In this regard, MIAT decreases the levels of miR-29c, resulting in an increase in lysyl oxidase-like 2 (LOXL2), an enzyme that catalyzes the oxidative deamination of lysine and hydroxylysine residues in tropocollagen and tropoelastin [85]. LOXL2 is a highly expressed protein in hepatic IR in response to lipotoxicity. Circulating LOXL2 levels were increased in T2D patients with clinical fibrosis [86]. In contrast, a significant increase in MIAT has been reported in diabetic rats and endothelial cells exposed to high glucose concentrations. Additionally, MIAT overexpression in endothelial cells has been reported to upregulate VEGF levels by inhibiting miR-150-5p, which suggests that there is an interplay among MIAT, miR-150-5p, and VEGF to promote angiogenesis, which is related to metabolic diseases (Figure 3) [87].

Ying et al. reported that a reduction in miR-150 increases the plasma levels of proinflammatory cytokines such as TNF-α, IL-1β, and IL-6, with the subsequent activation of JNK; these events could participate in the development of IR [88]. Hepatic IR could be generated by the inhibition of MIAT, which leads to the overexpression of miR-145 and a significant reduction in the phosphorylation of AKT, affecting the PI3K/AKT pathway, which leads to IR and decreased cell viability [89,90]. miR-214 is another molecular target of MIAT [91]. Reduction in miR-214 is associated with the IR state in cellular or animal models and in obese patients [92]. As mentioned above, MIAT decreases the levels of miRNAs with anti-inflammatory functions. It has been suggested that a reduction in miRNAs linked to anti-inflammatory functions induces an inflammatory response and the subsequent activation of serine/threonine kinases, such as JNK, a negative regulator of insulin activity via the PI3K/AKT pathway [93]. Moreover, MIAT promotes the upregulation of FoxO1, and this effect is associated with the ceRNA activity of MIAT on miR-139-5p in hepatocytes [94] (Figure 3). Considering the above-mentioned studies, MIAT, as with other lncRNAs, could impact the development of IR by regulating important genes involved in immune response signaling pathways, a mechanism that has become increasingly recognized as important in the development of metabolic diseases, including IR.

### 3.5. Steroid Receptor RNA Activator (SRA)

SRA is a type of lncRNA that coordinates the functions of various transcription factors, enhances steroid receptor-dependent gene expression, and serves as a distinct scaffold for corepressor complexes. SRA functions as an RNA coactivator for several nuclear hormone receptors, including androgen, estrogen, progesterone, glucocorticoid, thyroid hormone, retinoic acid, and vitamin D receptors [95,96,97], and is predominantly cytoplasmic [95]. SRA is encoded by the SRA1 gene, which is located on the short arm of human chromosome 5q31.3. The SRA1 gene is unique in that it encodes RNA transcripts of different lengths and produces an mRNA that encodes a 236/237-amino-acid protein through alternative splicing [98].

Interestingly, SRA is highly expressed in white adipose tissue (WAT) and functions as a transcriptional coactivator of PPARγ in in vitro models, suggesting that adipogenesis and adipocyte function can be affected by the SRA/PPARγ axis. Moreover, SRA increases insulin-stimulated glucose uptake and the phosphorylation of the downstream targets AKT and FoxO1 in adipose tissue [97]. Furthermore, SRA1 knockout mice were observed to have improved insulin sensitivity, decreased liver fat, and increased AKT phosphorylation in WAT, liver, and gastrocnemius muscle [99]. In primary mouse hepatocytes, Chen et al. identified that SRA expression stimulated the insulin-induced phosphorylation of AKT, extracellular signal-regulated kinase 1/2 (ERK1/2), and FoxO1, which decreased when SRA expression was inhibited. The researchers hypothesized that SRA functions as a scaffold to recruit a repressive complex in the liver to suppress FoxO1 and PPARγ-mediated adipose triglyceride lipase (ATGL) transcription [100]. SRA regulates multiple signaling pathways, including SRA/PPARγ/ATGL, AKT/FoxO1, and TNF-α/JNK pathways [98]. These data indicate that SRA plays an essential role in insulin signaling and the regulation of β-oxidation.

### 3.6. HOX Transcript Antisense RNA (HOTAIR)

HOX transcript antisense RNA (HOTAIR) is a 2.2 kb lncRNA that is generated from the transcription of the antisense strand of the HOXC gene located on human chromosome 12q13.13. HOTAIR induces gene silencing by interacting with gene silencing machinery. The 5’-end of HOTAIR interacts with PRC2, and its 3’-end interacts with lysine-specific demethylase 1 (LSD1). The recruits of PRC2 and LSD1 by HOX transcript antisense RNA (HOTAIR) result in the methylation of histone H3 on lysine 27 and demethylation of H3 on lysine 4, which leads to gene silencing [101,102,103]. Moreover, HOTAIR is overexpressed in several types of cancer; in these pathological states, HOTAIR can be associated with the induction of metastasis, anticancer drug resistance, and epithelial–mesenchymal transition [104].

Li et al. investigated the role of HOTAIR in hepatic IR; their results showed that HOTAIR was overexpressed in patients with T2D compared with normal subjects, and animals on a high-fat diet or with diabetes showed the same upregulation of HOTAIR. Moreover, HOTAIR overexpression in HepG2 cells was associated with the elevated expression of PCK and G6PC genes and the subsequent reduction in glycogen levels and an increase in glucose delivery. In addition, the overexpression of HOTAIR in HepG2 cells decreased the phosphorylation levels of AKT and glycogen synthase kinase 3 beta (GSK3β) after insulin stimulation. Finally, the author suggested that overexpression of HOTAIR decreases the levels of SIRT1, and the overexpression of SIRT1 decreases the insulin-resistant state originating in HepG2 cells by the overexpression of HOTAIR [105].

HOTAIR overexpression in the serum of patients with diabetic retinopathy, along with a decrease in miR-20b expression, was reported. In addition, miR-120b was observed to be negatively correlated with poor glycemic control. HOTAIR was found to have complementary sequences to miR-20b [106]. Majumder et al. demonstrated that HOTAIR expression was consistently increased in the kidneys in both humans and mice with diabetes. Additionally, in glomerular podocytes exposed to high glucose levels, the authors identified high levels of HOTAIR expression, and this upregulation was dependent on the p65 subunit of the proinflammatory transcription factor NF-κB, which could be involved in the pathogenesis of diabetic kidney disease [107]. Similarly, HOTAIR overexpression was found in human retinal endothelial cells (HRECs) exposed to high doses of glucose. In addition, HOTAIR was localized in both the nucleus and perinuclear/cytosolic regions [108].

In contrast, Gao et al. found that HOTAIR was downregulated, whereas, as a target of HOTAIR, miR-34a was upregulated in the heart of diabetic mice and in high-glucose-treated H9c2 cells. Additionally, HOTAIR overexpression has been shown to protect against inflammation and oxidative stress induced by diabetes via the HOTAIR/miR-34a/SIRT1 axis [109]. In another study, the downregulated expression of HOTAIR was identified in both myocardial tissue and serum from patients with diabetic cardiomyopathy compared to patients with diabetes without cardiomyopathy and healthy controls. Interestingly, no significant differences were found in HOTAIR expression between patients with diabetes without cardiomyopathy and healthy subjects. In addition, both the expression level of HOTAIR and the phosphorylation of AKT were decreased in human cardiomyocytes exposed to higher doses of glucose. Thus, HOTAIR overexpression may upregulate the phosphorylation of AKT [110]. The above-described findings suggest an important role for HOTAIR in the pathogenesis of IR, diabetes, and diabetes-associated complications. However, much remains to be elucidated, and future research is needed to identify the effect of HOTAIR on the molecular mechanisms involved in metabolic diseases such as IR and diabetes.

### 3.7. Growth Arrest-Specific 5 (GAS5)

Growth arrest-specific 5 (GAS5), whose gene is located on chromosome 1q25.1, was first characterized as non-coding lncRNA by Coccia et al., followed by Raho et al. [111,112]. Interestingly, the locus that encodes GAS5 harbors several C/D box small nucleolar RNAs (snoRNAs), RNAs related to RNA methylation, and splicing and editing of mRNAs [113]. GAS5 was shown to participate in the cell cycle and modulation of insulin secretion in mouse pancreatic β-cells. Therefore, its participation in the maintenance of the identity and function of β-cells has also been implicated in the pathogenesis of T2D in mice [114]. However, in a study conducted in humans, GAS5 was one of the lncRNAs that showed a greater significant association in terms of its altered expression with T2D. However, this association was lost when adjusted for HOMA-IR and senescence markers, which means that this association could be closely linked to IR and downstream inflammatory signaling [35]. In contrast, in disorders associated with IR, such as polycystic ovary syndrome (PCOS), GAS5 expression was found to be downregulated: GAS5 expression was decreased in the serum of PCOS patients with IR, and the researchers found a negative correlation between HOMA-IR and GAS5 expression. GAS5 is a suggested biomarker for the diagnostic and predictive value of IR [115].

### 3.8. Gm15622

Gm15622 was significantly enriched in the liver and overexpressed due to an HFD. This overexpression favored the accumulation of lipids and liver steatosis in obese mice as a consequence of feeding an HFD. This lncRNA plays a vital role in the expression of lipid synthesis genes such as FAS. Gm15622 regulates SREBP-1c expression by competing for the binding of miR-742-3p in alpha mouse liver 12 (AML-12) cells. In other words, Gm15622 can act as a molecular sponge or ceRNA for miR-742-3p. Interestingly, metformin inhibits FAS and SREBP-1c expression by targeting Gm15622. Thus, metformin exerts its effect by regulating a network of genes involving Gm15622, miR-742-3p, and SREBP-1c [116]. However, further experimental studies are needed to demonstrate the existence of a gene orthologous to Gm28220202 in humans and to identify its role in IR and/or diabetes.

### 3.9. Regulator of Insulin Sensitivity and Autophagy (Risa)

Regulator of insulin sensitivity and autophagy (Risa) or AK044604 is a potential lncRNA identified in different tissues, including the heart, kidney, liver, lung, muscle, spleen, thyroid, and white adipose tissue. Risa is located at the 10qB4 region close to the locus of SIRT1, an essential regulator of insulin sensitivity, autophagy, and metabolism. Risa is a polyadenylated lncRNA, and its overexpression significantly reduced the insulin-induced phosphorylation level of the insulin receptor, AKT, and GSK3β, which suggests that the overexpression of Risa induces IR [117]. Although this example illustrates a potential lncRNA that participates in the induction of IR and related diseases, studies in patients with IR and T2D are necessary.

### 3.10. Downregulated Expression-Related Hexose/Glucose Transport Enhancer (DREH)

Downregulated expression-related hexose/glucose transport enhancer (DREH) was initially named for its downregulated expression by hepatitis B virus X protein (HBx). DREH is an lncRNA in HBx transgenic mice [118]. Although the role of this lncRNA has not been directly demonstrated in IR, some studies suggest that DREH could be involved. For example, metformin treatment has been shown to reduce IR [119]. DREH expression does not facilitate glucose uptake in adipocytes unless its expression is lowered [120]. Interestingly, treatment with metformin leads to the dysregulation of several lncRNAs, including the deregulation of DREH expression [121].

**Figure 4:**
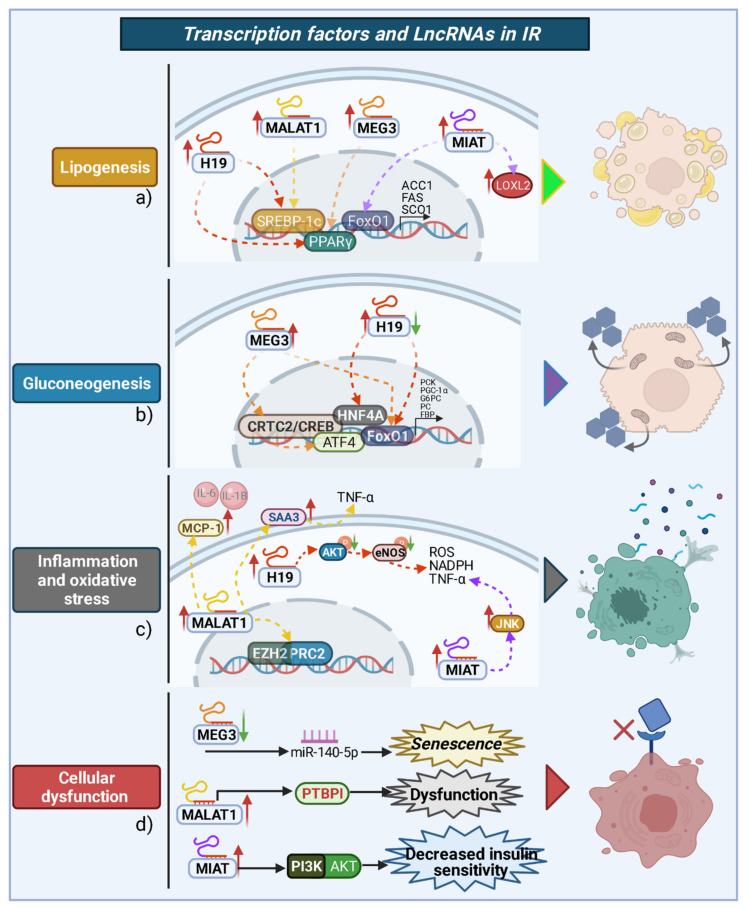
lncRNAs and transcription factors related to the development of IR. (**a**) Lipogenic activity is associated with elevated levels of H19, MALAT1, MEG3, and MIAT and their interaction with nuclear proteins, such as element-binding transcription factor 1c (SREBP-1c), proliferator-activated receptor gamma PPARγ, and forkhead box O1 (FoxO1), and in consequence, the expression of lipogenic genes is promoted, e.g., acetyl-CoA carboxylase 1 (ACC1), fatty acid synthase (FAS), and cytochrome C oxidase (SCO1), as well as other proteins such as lysyl oxidase-like 2 (LOXL2) by MIAT, which leads to the accumulation of lipids. (**b**) MEG3 upregulation activates CREB-regulated transcriptional coactivator (CRTC2), which in turn activates (cAMP response element-binding protein) CREB by binding to activating transcription factor 4 (ATF4) and FoxO1; in addition, hepatocyte nuclear factor 4 alpha (HNF4A) and FoxO1 are activated by H19 overexpression, which induces gluconeogenesis. In contrast, H19 downregulation promotes the transcription of FoxO1 and the gluconeogenic genes. (**c**) MALAT1 overexpression induces the expression of zeste polycomb homolog 2 (EZH2)/polycomb repressive complex 2 (PRC2), monocyte chemoattractant protein-1 (MCP-1), and serum amyloid antigen 3 (SAA3), which in turn promote the elevation of interleukins 6 and 1β, and tumor necrosis factor-alpha (TNF-α). Additionally, the expression of H19 induces the dephosphorylation of protein kinase B (AKT) and nitric oxide synthase (eNOS), and adenine dinucleotide phosphate oxidase (NADPH) and TNF-α levels increase as a result of the expression of c-Jun-N-terminal kinases (JNK) due to MIAT. (**d**) MEG3 downregulation promotes miR-140-5p expression, which is related to cellular senescence. Overexpression of MALAT1 activates polypyrimidine tract-binding protein 1 (PTBPI), which is involved in pancreatic β-cell dysfunction. MIAT downregulation affects the phosphatidylinositol 3-kinase/protein kinase B (PI3K/AKT) pathway signaling, decreasing insulin sensitivity and cell viability.

As previously described, several mechanisms have been reported to explain the key role of lncRNAs in the development of IR. However, studies have identified several molecular targets for only a few lncRNAs described in this manuscript. On the other hand, the mechanisms linked to molecular, cellular, and biochemical actions of lncRNAs vary according to the tissue, biological species, and the severity of IR (Figure 4). Table 1 summarizes studies that researched the association between the aberrant expression of other lncRNAs and their main molecular targets during IR or associated pathologies.

## 4. Conclusions and Future Perspectives

Insulin resistance is a multifactorial disease associated with metabolic syndrome and is considered a risk factor for the development of T2D, dyslipidemias, obesity, and cardiovascular diseases. Environmental risk factors, such as a sedentary lifestyle, stress, and malnutrition, and genetic factors, such as single-nucleotide polymorphisms (SNPs) and copy number variation, can contribute to the development of IR. However, in recent years, non-coding RNAs, including miRNAs and lncRNAs, have acquired increased relevance, the latter being the most clinically important because they are attributed various functions, such as acting as master regulators of different genetic, epigenetic, and metabolic processes, under normal physiological conditions and in metabolic disorders related to diseases. Various authors have associated lncRNAs, such as MIAT, MALAT1, H19, MEG3, and HOTAIR, with inflammatory and lipogenic processes that can lead to IR development through interactions with mRNAs, miRNAs, transcription factors, and proteins. However, a direct relationship between lncRNAs and the insulin signaling pathway or glucose metabolism has not yet been identified. Most of the presented results are from in vivo and in vitro models, and studies in patients need to be conducted to demonstrate the utility of these molecules by generating and implementing panels of the lncRNAs described here. In conclusion, it is essential to continue conducting research that clarifies the direct involvement of lncRNAs in the molecular mechanisms that contribute to IR and their molecular interaction networks that regulate insulin signaling pathways. In addition, it is necessary to demonstrate the usefulness of these biomolecules as potential biomarkers for the diagnosis and prognosis of the development of IR, as well as their potential application as therapeutic targets.

## Figures and Tables

**Table 1:** Roles of other lncRNAs in molecular mechanisms associated with insulin resistance and related diseases.

LncRNAs	Pathology	Model	Tissue	Main Target	Regulating during IR	Reference
NONMMUT008655.2	IR	High-fat-diet-induced IR mice	Liver	Regulation of SOC- S3 via miR-133c, miR-3569-5p, miR-504-3p, and miR-7076-5p	Upregulated in IR. Resveratrol improves insulin sensitivity by downregulating this lncRNA.	[122]
ASMER-1 (ENSG00000235609.4) ASMER-2 (CATG00000111229.1)	Obesity and IR	Human	White adipose	PPARγ	Silencing of ASMER-1 and ASMER-2 decreases the expression of adipogenic genes and reduces the triglyceride accumulation in adipocytes.	[123]
lncLSTR	Without	Mice	Liver	TDP-43/Cyp8b1	Depletion of *lncLSTR* leads to reduced triglyceride levels.	[124]
ANRIL (CDKN2B-AS1)	Diabetes	HRECs cells exposed to high levels of glucose and diabetic mice	Retinal	PRC2/VEGF	High levels of glucose promote the overexpression of ANRIL. ANRIL inhibition promotes glucose-induced VEGF overexpression.	[125]
CTD-2517M22.14	Hepatic IR	IR-HepG2 cells	Liver	miR-346; miR-1306-5p; miR-18a-3p; miR-1914-5p_R_1/SCD	These lncRNAs positively regulate MAPK, linked to inflammation and IR. The reduction in ROS with a subsequent decrease in the relative expression of all lncRNAs might ameliorate inflammation.	[126]
CTD-2600O9.2				miR-486-3p/DUSP5		
CTD-3014M21.1				miR-324-5p, miR-2478_L-1/SCD		
LAMA5-AS1				miR-18a-3p; miR-516b-5p; miR-763-p3/SCD		
HOXB-AS3				PC-5p-36422_6/SCD		
RP5-1057I20.5				miR-23b-5p, miR-185-3p, miR-642a-5p/SCD		
MIR4435-2HG				miR-149-3p_L; miR-1304-3p; miR-452-5p/PLK3		
ENSMUST00000160839	IR	Palmitic acid-induced IR C2C12 cells	Myotubes	PDK-4	PDK4 upregulation has been related to IR and was positively correlated with ENSMUST00000160839.	[127]
ENST00000550337.1	Pre-diabetes and T2D	Human	Whole blood	Undescribed	ENST00000550337.1 could be a potential diagnostic biomarker for pre-diabetes and T2D.	[35,128]
PLUTO CDKN2BAS1 LincRNA-p21 XIST PANDA NBR2 LET SALRNA1 THRIL	T2D	Human	PBMC	Undescribed	The majority of the lncRNAs were positively correlated with poor glycemic control, IR, transcriptional markers of senescence, inflammation, and HDAC3 and negatively associated with telomere length.	[35]
p5549	Obesity	Human	Whole blood (in addition, 4 blood components) and adipose tissue	TDG	The 3 lncRNAs were negatively correlated with BMI, waist circumference, and waist-to-hip ratio. Only the circulating level of p19461 was negatively associated with IR. TDG is involved in DNA repair. CHCHD3 regulation could protect mitochondrial function, accelerating fat oxidation and reducing obesity.	[129]
p21015				Undescribed		
p19461				CHCHD3		
lncSHGL	Obesity and IR	High-fat-diet-induced IR mice	Primary mouse hepatocytes	hnRNPA1/CALM/Akt	lncSHGL induced Akt activation via lncSHGL/hnRNPA1/CALM pathway. Downregulation of lncSHGL promotes IR and fatty liver.	[130]

IR: Insulin resistance; SOCS3: Suppressor of cytokine signaling 3; PPARγ: Peroxisome proliferator-activated receptor gamma; TDP-43: Transactive response DNA binding protein 43; Cyp8b1: Cytochrome P450 Family 8 Subfamily B Member 1; HRECs: Human retinal endothelial cells; PRC2: Polycomb repressive complex 2; miR: micro-RNA; VEGF: Vascular endothelial growth factor; SCD: Stearoyl-CoA desaturase; DUSP5: Dual specificity phosphatase 5; PLK3: Polo-like kinase 3; ROS: Reactive oxygen species; PDk4: Pyruvate Dehydrogenase Kinase 4; PBMC: Peripheral blood mononuclear cells; HDAC3: Histone deacetylase 3; TDG: Thymine-DNA glycosylase; CHCHD3: Coiled-Coil-Helix-Coiled-Coil-Helix Domain-Containing Protein 3; hnRNPA1: Heterogeneous Nuclear Ribonucleoprotein A1; CALM: Calmodulin; Akt: Protein kinase B.

## Data Availability

Not applicable.

## Abbreviations

ACC1	Acetyl-CoA carboxylase 1
AGEs	Advanced glycation end products
AKT	Protein kinase B
AMI	Acute myocardial infarction
AML-12	Alpha mouse liver 12 cells
AMPK	AMP-activated protein kinase
ATF4	Activating transcription factor 4
ATGL	Adipose triglyceride lipase
cAMP	Cyclic adenosine monophosphate
ceRNA	Competing endogenous RNA
CoA	Coenzyme A
CPT1b	Carnitine palmitoyltransferase 1b
CREB	cAMP responsive element-binding protein
CRTC2	Regulated transcription coactivator-2
CVD	Cardiovascular diseases
DAG	Diacylglycerol
DNMT	DNA methyltransferase
DREH	Hexose/glucose transport enhancer
DUSP27	Dual-specificity phosphatase 27
EGR2	Early growth response protein 2
eNOS	Nitric oxide synthase
EPCs	Endothelial progenitor cells
ERK1/2	Extracellular signal-regulated kinase 1/2
EZH2	Enhancer of zeste polycomb repressive complex 2
FAS	Fatty acid synthase
FBP	Fructose-1,6-biphosphatase
FFA	Free fatty acids
FGF	Fibroblast growth factor
FoxO1	Forkhead box O1
G6PC	Glucose-6-phosphatase catalytic
GAS5	Growth arrest specific 5
GK	Glucokinase
GLUT4	Glucose transporter type 4
GSK3β	Glycogen synthase kinase 3 beta
HBx	Hepatitis B virus X
HFD	High-fat diet
HGP	Hepatic glucose production
HMGB1	High-mobility group box 1
HNF4A	Hepatocyte nuclear factor 4 alpha
hnRNPA1	Heterogeneous nuclear ribonucleoprotein A1
HOMA-IR	Insulin resistance- homeostatic model assessment
HOTAIR	HOX transcript antisense RNA
HRECs	Human retinal endothelia cells
IGF	Insulin-like growth factor
IKK	Inhibitor of nuclear factor kappa
IL	Interleukin
INSR	Insulin receptor
Let-7	Lethal-7
LOXL2	Lysyl oxidase like 2
IR	Insulin resistance
IRS	Insulin receptor substrate
JNK	c-Jun-N-terminal kinase
KSRP	K-homology splicing regulatory protein
LSD1	Lysine-specific demethylase 1
MALAT1	Metastasis associated lung adenocarcinoma transcript 1
MAPK	Mitogen-activated protein kinase
MCP-1	Monocyte chemoattractant protein-1
MEG3	Maternally expressed gene 3
MIAT	Myocardial infarction associated transcript
MLXIPL	MLX-interacting protein-like
mTOR	Mammalian target of rapamycin
mTORC1	mTOR complex 1
NADPH	Nicotinamide adenine dinucleotide phosphate oxidase
NF-κB	Nuclear factor kappa B
PC	Pyruvate carboxylase
PCK/PEPCK	Phosphoenolpyruvate carboxykinase
PCOS	Polycystic ovary syndrome
PDK1	Pyruvate dehydrogenase kinase 1
PDX1	Pancreatic and duodenal homeobox 1
PGC-1α	Peroxisome proliferator-activated receptor gamma coactivator 1-alpha
PI3K	Phosphatidylinositol 3 kinase
PK	Phosphokinase
PPAR	Peroxisome proliferator-activated receptor
PRC2	Polycomb repressive complex 2
PTBP1	Polypyrimidine tract-binding protein 1
Risa	Regulator of insulin sensitivity and autophagy
ROS	Reactive oxygen species
SAA3	Serum amyloid antigen 3
SAH	S-adenosylhomocysteine
SAHH	S-adenosylhomocysteine hydrolase
SAT	Subcutaneous adipose tissue
SCO1	Cytochrome C oxidase
SFA	Saturated fatty acids
SIRT1	Sirtuin 1
SRA	Steroid receptor RNA activator
SREBP-1c	Sterol regulatory element-binding transcription factor 1c
T2D	Type 2 diabetes
TG	Triglycerides
TGF-β	Transforming growth factor-beta
TNF-α	Tumor necrosis factor-α
UPR	Unfolded protein response
VASH1	Vasohibin-1
VAT	Visceral adipose tissue
VEGFA	Vascular endothelial growth factor-A
WAT	White adipose tissue
